# Highly Effective Flame-Retardant Rigid Polyurethane Foams: Fabrication and Applications in Inhibition of Coal Combustion

**DOI:** 10.3390/polym11111776

**Published:** 2019-10-29

**Authors:** Liancong Wang, Benjamin Tawiah, Yongqian Shi, Suncheng Cai, Xiaohui Rao, Chuan Liu, Ye Yang, Fuqiang Yang, Bin Yu, Yuntao Liang, Libi Fu

**Affiliations:** 1 State Key Laboratory of Fire Science, University of Science and Technology of China, 96 Jinzhai Road, Hefei 230026, China; wangliancong-0829@163.com; 2 State Key Laboratory of Coal Mine Safety Technology, CCTEG Shenyang Research Institute, Fushun 113122, China; 3 Institute of Textiles and Clothing, Hong Kong Polytechnic University, Hong Kong 999077, China; tawiahb@gmail.com; 4 College of Environment and Resources, Fuzhou University, 2 Xueyuan Road, Fuzhou 350116, China; dandy_cai@163.com (S.C.); Rxh123456789m88@163.com (X.R.); liuchuanll@163.com (C.L.); ys19750873@163.com (Y.Y.); fqouyang82@163.com (F.Y.); 5 Centre for Future Materials, University of Southern Queensland, Toowoomba QLD 4350, Australia; 6 College of Civil Engineering, Fuzhou University, 2 Xueyuan Road, Fuzhou 350116, China; fulibi@fzu.edu.cn

**Keywords:** Polyurethane foam, intumescent flame-retardant, coal combustion, expandable graphite, phosphorus

## Abstract

The extemporaneous combustion of coal remains a major threat to safety in coal mines because such fire accidents result in casualties and significant property loss, as well as serious environmental pollution. This work proposed the fabrication of flame-retardant rigid polyurethane foam (RPUF) containing expandable graphite as char expander/sealant with melamine phosphosphate and 2-carboxyethyl (phenyl)phosphinic acid as char inducer and radical trapping agents. The as-prepared RPUF successfully inhibited coal combustion by forming thermally stable high graphitic content expandable intumescent char sealing over the coal. The RPUF achieved UL-94 V-0 rating in addition to significant reductions in peak heat release, total heat release, and CO and CO_2_ yields. The external and the internal residual char structure was studied by X-ray photoelectron spectra, Raman spectroscopy, and real-time Fourier transform infrared spectra techniques, and a flame-retardant mode of action has been proposed. This work provides important insight into a facile fabrication of highly efficient and economical flame-retardant RPUF to inhibit the spontaneous combustion of coal.

## 1. Introduction

Polyurethanes (PUs) are some of the most versatile materials of the 21st century with wide applications in upholstered furniture, wall insulation, roofs, appliances, medical devices, footwear, coatings, adhesives, and sealants. Its elastomeric derivatives are used on floors and automotive interiors [1,2,3]. Among the several derivatives of PU, rigid polyurethane foam (RPUF) is the more attractive due to its physico-chemical properties such as low thermal conductivity and density, high abrasion resistance, and good shock absorption [4,5]. Despite the attractive properties of RPUF, it is a highly combustible polymer with fast flame spread and high heat release [6,7]. As a result, the urge to improve the flame-retardant properties of RPUF has received significant attention over the last few decades [8,9,10,11].

Though halogen containing flame retardants (FRs) are the most promising additives for RPUF, their adverse health effect far outweighs their perceived flame-retardant significance [9,12,13,14,15]. Therefore, non-halogenated FRs containing significant amount of phosphorus-nitrogen (P/N) has been investigated as intumescent flame retardants (IFRs) for RPUFs [16,17]. The P/N derivatives provide excellent fire resistance with less smoke formation and lower toxicity when combined with other carbonizing and foaming agent such as pentaerythritol, sorbitol, and melamine, etc. [18,19,20]. The IFR system is preferred especially for RPUFs because of their ability to undergo extreme expansion and form protective charred layers that can act as physical barriers to protect the underlying RPUF from the flame [21]. Owing to these unique advantages, several novel IFR systems have been proposed to significantly improve flame retardancy for RPUFs by integrating the functional components of IFR (acid source, carbonization agent, and blowing agent) into a single molecule [18,22,23]. These types of FRs display excellent flame-retardant performance. However, the ensuing char is usually not compact enough to significantly reduce smoke and toxic gas evolution [22,23,24]. As a result, many studies have proposed the combination of conventional IFR with expandable graphite (EG) to enhance the char expansion and sealing effect to surmount the problem of excessive smoke and toxic fumes leakage during combustion [25,26,27]. Based on this supposition, a novel IFR using EG and diethyl ethylphosphonate with organically modified nanoclay and layered double hydroxide was designed to achieve significantly reduced smoke and toxic fume production during combustion [28]. In a similar fashion, a liquid-based phosphorus-containing polyol in conjunction with EG was used to substantially reduce total smoke release (TSR) and CO production [29]. However, these FRs have obvious drawbacks for the inhibition of coal fires where a more thermally stable high sealant IFRs system is required. IFRs forming loose char structures upon burning will allow oxygen to slip through the micro holes and thus cause smoldering combustion over long period of time. Therefore, it is necessary to design a suitable IFR for RPUFs with a high sealant effect to overcome the aforementioned problems. The various techniques available for averting the spontaneous combustion of coal comprise spraying an inhibitor, chemical fog, gel fog, or inert gel fog onto the surface [30,31]. Others include grouting, pressure equalization, injection of inert gases, gels, foams (including three-phase foams) and air sealing using composite clay slurries [32,33,34]. Methods such as the use of inorganic foams and other polymeric materials have also been suggested [35,36]. Most of these techniques are plausible but at the same time are quite complicated and expensive. Moreover, for the techniques that involve the use of inert gases, the gases often diffuse easily from the injection area due to leakages, which are sometimes difficult to control. Besides these challenges, three-phase foams are difficult to cure completely, and have a short life span of 8–12 h, beyond which the foams become unstable. In addition, exothermic curing can trigger coal combustion easily. Given the obvious drawbacks of the coal inhibition systems described above, an intelligent self-foaming hydrogel filled with EG from copolymerization of corn straw, 2-acrylamide-2-methylpropanesulfonic acid (AMPS), and acrylic acid (AA) was produced and applied to inhibit the spontaneous coal combustion [30]. The hydrogel exhibited good thermal stability, and good surface coverage area and degree of swelling at high temperature. This permitted reduction in CO production. Similarly, a multiphase foam gel composed of fly ash (FA), foaming agent, thickener, crosslinker, and nitrogen, formed by physical and mechanical stirring has been investigated [37]. The study indicated that the multiphase foam was able to reduce the spontaneity of coal combustion to some level with some acceptable reductions in CO production. However, TSR and other gas production was not reported.

Although RPUF have been much studied, very little attention has been directed toward the potential flame-retardant effect of stoichiometric variations of char sealant combined with FA and other FRs containing significant amounts of N and P such as 2-carboxyethyl (phenyl)phosphinic acid (CEPPA), and melamine phosphate (MP). EG possess the inherent ability to expand more than 200 times its original size, and when combined with FA forms a compact char with an excellent sealing effect in PU.

The effect of the stoichiometric loadings of EG, CEPPA, MP, and FA as highly effective flame-retardant RPUF on the inhibition of spontaneous coal combustion has been studied. The EG serves as char expander with FA as sealant and blowing agent. In addition, radical trapping by phosphorus radicals from MP and CEPPA occurs. The RPUF can be used as insulation in electronic materials. It can also be used to prevent the common problem of spontaneous coal combustion in underground coal mines and coal heaps before shipping or for preventing fires in abandoned mines.

## 2. Materials and Methods

### 2.1. Raw Materials

**Figure 1:**
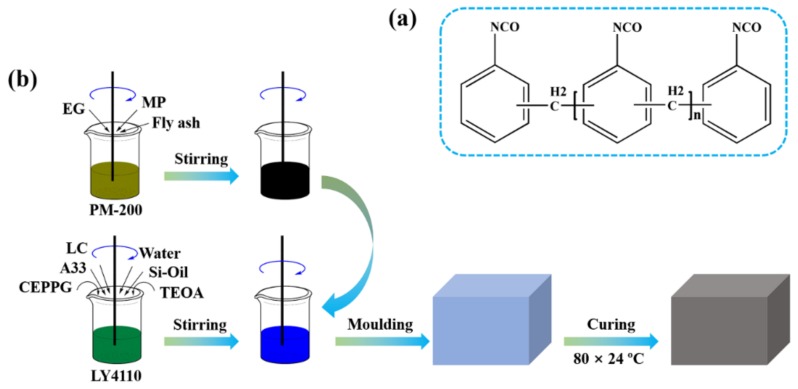
(**a**) Chemical structure of PAPI; (**b**) Schematic diagram for fabrication of flame-retardant RPUF composites.

Polyether polyol (LY-4110, –OH content: 430 mg KOH g^−1^, viscosity at 25 °C: 2500 mPa s), polyaryl polymethylene isocyanate (PAPI) (PM-200, NCO: 30.5–32%, viscosity at 25 °C: 150–250 mPa s) (See Figure 1a), dibutyltin dilaurate (LC), triethylenediamine (A33), and silicone surfactant (Si–Oil) were graciously provided by Jiangsu Luyuan New Materials Co., Ltd. (Nantong, China). Ethylene glycol, triethanolamine (TEOA), melamine, phosphoric acid (≥85%) were all obtained from Sinopharm Chemical Reagent Co., Ltd. (Shanghai, China). EG was purchased from Qingdao Tianhe Graphite Company (Particle size: 80 meshes, Qingdao, China). CEPPA was obtained from Jinan Kerry Flame Retardant Technology Co., Ltd. (Jinan, China). FA (particle size: 200–300 meshes) was supplied by Rong Changsheng Water Purification Material Co. Ltd. (Zhengzhou, China). Ultrapure water (18.2 MΩ cm^−1^) was obtained from a Milli-Q ultrapure system (Zhengzhou, China). CEPPG was obtained by the esterification reaction of CEPPA and ethylene glycol according to the previous report [38]. MP was synthesized by the salt forming reaction of melamine and phosphoric acid [39]. The brown coal was provided by Liujia Coal Mine, Inner Mongolia Pingzhuang Energy Co., Ltd. (Chifeng, China). Other chemicals were used as obtained.

### 2.2. Preparation of Flame-Retardant Rigid Polyurethane Foams

Flame-retardant RPUF composites were fabricated according on a laboratory scale (See Figure 1b), and their formulation compositions are listed in Table 1.

### 2.3. Inhibition of Coal Combustion

In the coal combustion experimental test, 200 g of coal sample was put in a stainless steel medical square plate with the size of 16 × 16 cm^2^. Then, the equivalent flame-retardant RPUF composites were formed on different coal samples. After that, the flame-retardant RPUF composites which had covered coal samples were dried in ambient environment for 24 h or under 80 °C for 24 h. The spray gun filled with butane gas was used to cauterize the RPUF composites covered coal samples for 5 min.

### 2.4. Instruments and Measurements

Thermogravimetric analysis (TGA) of the flame-retardant RPUF composites was conducted using a Q5000 thermal analyzer (TA Co., New Castle, DE, USA) in the range of 50–750 °C at a heating rate of 20 °C min^−1^ in air atmosphere [40]. UL-94 vertical burning test. The vertical burning test was conducted by a CZF-II horizontal and vertical burning tester (Jiang Ning Analysis Instrument Company, Nanjing, China). The specimens used were 127 × 13 × 10 mm^3^ according to ASTM D3801-2010. The combustion behaviors of the flame-retardant RPUF composites were evaluated by a cone calorimeter (TESTech, Suzhou, China) according to ISO 5660-1 under the heat flux of 35 kW m^2^. The specimen with size of 100 × 100 × 25 mm^3^ was placed in an aluminum foil. The morphologies of the fracture surfaces of char residues of the composites were investigated by SEM (Nova NanoSEM 230, FEI CZECH REPUBLIC S.R.O., Brno, Czech Republic). Each specimen was sputter coated with a gold layer before observation. Raman spectroscopy was recorded on a Renishaw Invia Raman Microscope (Invia Reflex, Renishaw Invia, UK) using a 532 nm argon ion laser at a spectroscopic resolution of 1.2 cm^−1^ for each sample. X-ray photoelectron spectrometer (XPS) spectra were recorded using a Thermo Fisher Scientific K-Alpha spectrometer (Waltham, MA, USA) employing a monochromatic Al Kα X-ray source (h*ν* = 1486.8 eV). The real-time Fourier transform infrared (RTIR) spectra of obtained samples were recorded at a linear heating rate of 20 °C min^−1^ from room temperature to 600 °C.

## 3. Results and Discussion

### 3.1. Thermal Degradation Behavior Analysis

**Figure 2:**
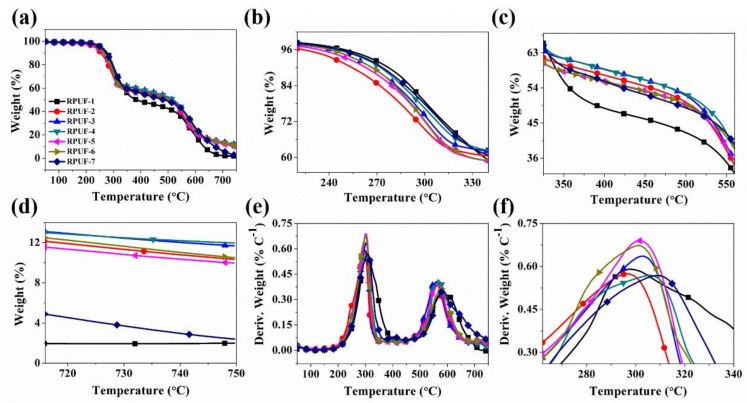
(**a**–**d**) TG and (**e**,**f**) DTG curves of RPUF and its composites under air condition.

The thermo-oxidative properties of RPUF and its flame retarded composites were studied under air, and the related results are shown in Figure 2 and Table 2. Three major thermal transitions are observed for pure RPUF (RPUF-1) and its flame retarded composites (FRCs) (See Figure 2b). It is observed that pristine RPUF remains thermally stable during the first thermal event that occurs around 210–320 °C compared to the FRCs. The small mass loss (~3.5%) in this region is associated with the dissociation of biuret and allophanate, which is the thermally weakest link in the RPUF network into their precursor materials [41]. During this process, all the units that connect the polyether chains regenerate into their respective precursor compounds made up of isocyanate, hydroxyl compounds, and various forms of amines. The early mass loss for the FRCs (See Figure 2e,f) is ascribed to the evaporation of adsorbed/crystal water in FA resulting from its foaming properties and the dissociation of the –OH groups in the polyether polyol followed by the possible release of the phosphoxide radicals (PO•) from CEPPG. In the second thermal transition which occurs around 320-560 °C, the FRCs (RPUF-2~RPUF-7) remain thermally stable probably due to the catalytic charring effect of the derivatives generated by MP and CEPPG on the matrix. In contrast, the pristine RPUF undergoes significant mass loss in this region, as shown in Figure 2c and Table 2 due to the absence of FA, CEPPG, and MP.

The third thermal event occurring around 560–750 °C is principally due to the conversion of the depolymerized isocyanate unites, polyols, EG, and the phosphorus contents into char residues in the condensed phase [42]. The formed char undergoes further oxidation which reduces its quantity with the increasing heat supply. Eventually, at 750 °C, the FRCs (RPUF-2~RPUF-6) show significantly higher char residue compared to the RPUF-1 and RPUF-7, primarily due to the simultaneous presence of FA, MP, and CEPPG.

To understand the influence of MP (which is the main source of phosphorus) on the charring predisposition of RPUF, RPUF-7 was formulated without MP, and obviously exhibits lower char content (See Table 2) despite the presence of FA and CEPPG. It is observed that as the content of phosphorus (P) increases in RPUF (See Figure 2d and Table 2), the char residue increases correspondingly, which affirms the charring and flame-retardant effect of MP in RPUFs composition. The char formation by MP is principally due to the flame-retardant mechanism of P element in the condensed phase together with the increasing content of EG especially in RPUF-3 and RPUF-4 during the endothermic degradation. The phenomenon suggests that the combination of MP and higher EG content promotes char formation as shown in Table 2. EG known to maintain its integrity in the flames provides better fire protection because its flame-retardant action is mainly in the condensed phase as a smoke/toxic suppressant and a char sealant [43]. The formation of intumescent char is very essential for achieving a desirable flame-retardant effect especially for RPUFs.

### 3.2. Flame-Retardant Performance Evaluation

#### 3.2.1. Vertical Combustion Testing

**Figure 3:**
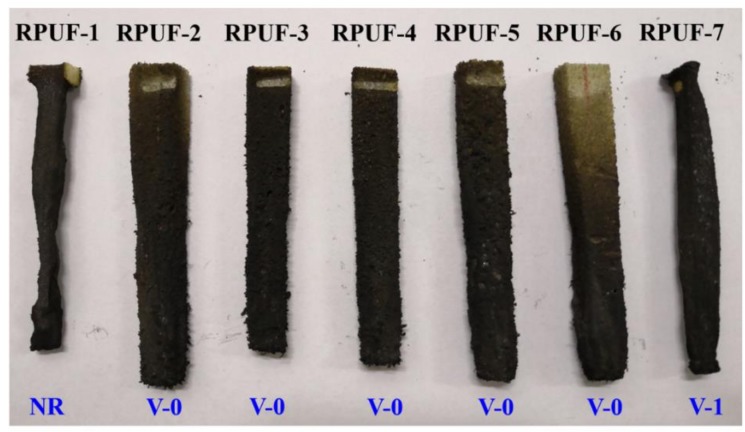
Digital photographs of char residues for RPUF and its composites after vertical burning tests.

Flame-retardant property of RPUFs was investigated by the UL-94 vertical burning test, and the results are shown in Figure 3 and Table S1. The pristine RPUF has no rating because the sample burns fiercely over a prolonged period of time with heavy dripping leaving little residue.

Upon introducing CPPEG, FA, and MP into the RPUF i.e., RPUF-2~RPUF-6, the RPUFs can pass the V-0 rating, and the burning time reduces significantly without dripping. The length of RPUF-5 and RPUF-6 remain unaffected despite the prolonged torching time due to the higher P content in those samples (See Table 1). This phenomenon is due to the flame-retardant action of P element and EG in the condensed phase during combustion. It is obvious that the flame burns only along the surface of RPUF samples and thus creates char residue which essentially protects the unburnt inner core of the RPUF from the fire. The char formed on the surface of the RPUFs serves as a complete barrier/blockade to prevent the flame further access into the unburnt inner core. Based on the action, the flame can be self-extinguished after burning the fluffy surface layers of the foam only. The presence of EG, FA, and MP plays a critical role in this phenomenon. Unlike RPUFs containing EG, FA, and MP, RPUF-7 has a prolonged burning time with the fast burning (See Table S1). This phenomenon confirms the importance of EG, FA, and MP in the fire retardancy of RPUF. Moreover, it is found that the samples containing higher P element i.e., RPUF-5 and RPUF-6 still have part of the fluffy surface intact. This further underscores the important role of MP in the fire safety of RPUF composites.

#### 3.2.2. Cone Calorimeter Testing

**Figure 4:**
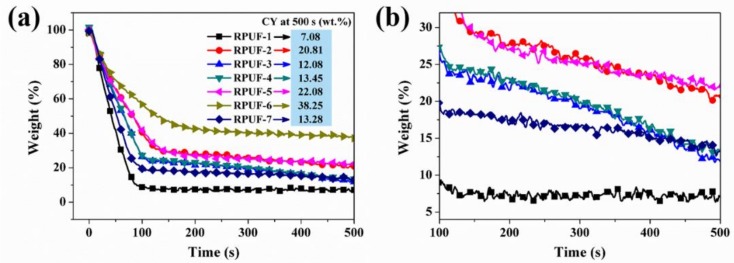
(**a**) Weight loss and (**b**) magnified weight loss curves of RPUF and its composites during combustion.

Figure 4a,b present the weight loss of RPUF-1~RPUF-7 obtained from the cone calorimeter test. The mass loss of pristine RPUF and flame retarded RPUF starts around the same time (4–6 s); however, the RPUF-1 has higher and faster mass loss rate with relatively lower char residue (7.08 wt %) at the end of the burning (500 s). RPUF-2 containing significant amount of EG and higher stoichiometry quantity of MP shows lower degradation rate, resulting in higher char residue (See Table 1 and Figure 4a).

To understand the influence of EG and MP on the charring behavior of the composites, the content of EG is increased to 18 wt % and 20.25 wt % in RPUF-3 and RPUF-4, respectively, while that of MP is reduced to 9 wt % and 6.75 wt % in the same samples accordingly (See Table 1). It is evident that the RPUF-3 and RPUF-4 show higher degradation rate compared to RPUF-2. Similarly, RPUF-3 and RPUF-4 have lower char residues of 12.1 wt % and 13.5 wt % respectively compared to RPUF-2 (char residue of 20.8 wt %) (See Figure 4a,b). On the other hand, when the content of EG in RPUFs decreases to 6.75~9.00 wt % with a corresponding increment of MP to 18.00~20.25 wt % (RPUF-5 and RPUF-6), the mass loss rate is lower with a correspondingly higher char residue of 22.80~38.25 wt %, which reinforces the important contribution of MP to char expansion and compactness.

The P content of RPUF samples is recorded in Table 1. It is observed that the char formation and the flame-retardant efficiency of the system are directly related to the quantity of P-containing species in the RPUF system. This phenomenon discloses the significance of MP in the char formation and the flame retardancy of RPUF composites. MP acts as blowing agent and char inducer in the gas phase, and predominantly char inducer in the condensed phase due to the high N and P content.

To evaluate the contribution of phosphine in CEPPG to the char formation of the composites, RPUF-7 was fabricated without EG, FA, and MP. Although the mass loss rate of RPUF-7 declines marginally compared to RPUF-1, improvement in char formation is ca. 48% as compared to the pristine RPUF, which further confirms the significant contribution of P to char formation in RPUF composites.

**Figure 5:**
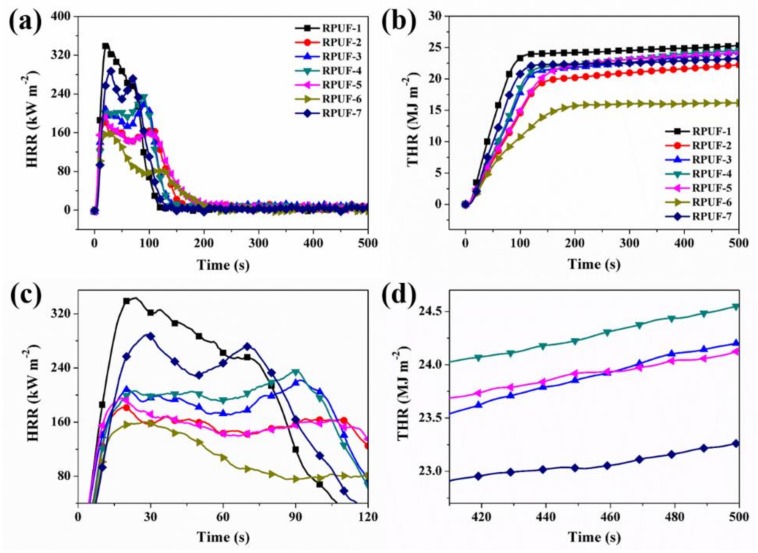
(**a**,**c**) HRR and (**b**,**d**) THR curves of RPUF and its composites.

Figure 5a–d shows the heat release rate (HRR) and total heat release (THR) curves of RPUF samples obtained from the cone calorimeter testing. The peak of HRR (PHRR) and THR of RPUF-2 are reduced by 46% and 12%, respectively, whereas for RPUF-3 and RPUF-4, the values of HRR are decreased by approximately 35.4% and 35.2%, respectively. Also, the values of THR decline by 4.7% and 3.1%, respectively. However, when the MP loading is increased to 18.00~20.25 wt % with a corresponding decrease in EG loading to 6.75~9.00 wt % in the same samples (RPUF 5 and RPUF 6), the values of PHRR increase to 42.5%~52.8%, as shown in Figure 5a,c, and Table 3. It is noted that the RPUF-6 shows significantly reduced THR by 35.8% (See Figure 5b,d and Table 3). In the case of RPUF-7, the PHRR is only reduced by 14.7%, and the THR is decreased marginally, due to the absence of EG, FA, and MP. The analysis of HRR and the THR results indicates the flame retardancy of RPUF composites can be enhanced even more significantly by controlling the ratios of MP to EG loadings.

**Figure 6:**
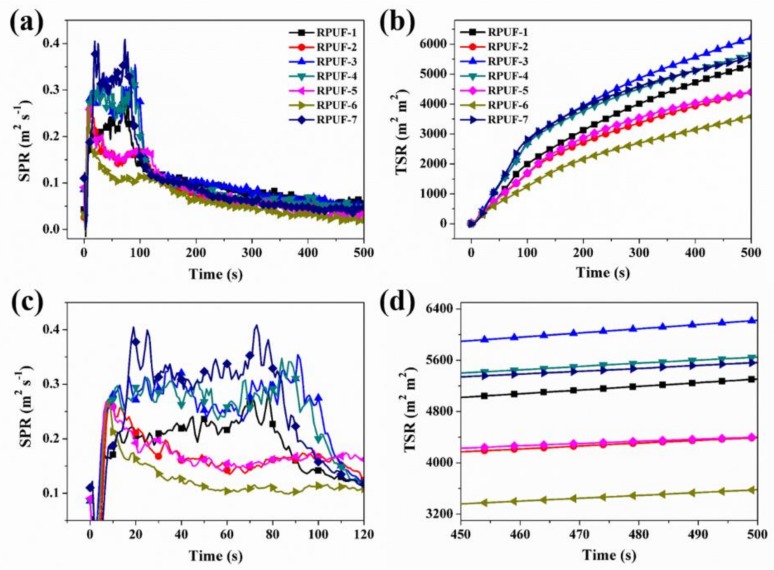
(**a**,**c**) SPR and (**b**,**d**) TSR curves of RPUF and its composites.

The smoke production rate (SPR) and TSR curves obtained from the cone calorimeter testing are shown in Figure 6a–d. Similar to the HRR curve, the smoke production happens between 4 s and 120 s. Relatively lower SPR is observed for RPUF-2, RPUF-5, and RPUF-6, whereas, higher SPR is visible for other RPUF samples, especially RPUF-7 (See Figure 6a,c). In a similar vein, the RPUF-6 has the lowest smoke release, while RPUF-3 achieves the highest TSR, as presented in Figure 6b,d and Table 3. The increased TSR for the RPUF-3 will result in reduced visibility and respiratory irritation as well as shortness of breath to occupants of buildings and firefighters during fire accidents. Fortunately, RPUF-2, RPUF-4, and RPUF-6 can reduce this occurrence due to obvious reductions recorded.

**Figure 7:**
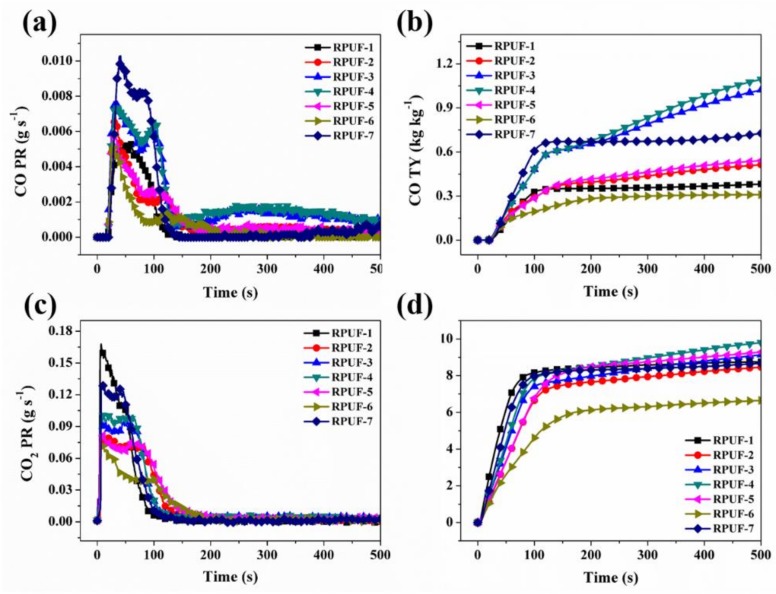
(**a**) CO PR, (**b**) CO TY, (**c**) CO_2_ PR and (**d**) CO_2_ TY curves of RPUF and its composites.

Besides the particulate smoke matter generated during fire accidents, CO, CO_2_, HCN, and other evolved gases are produced during the combustion of RPUF. The release of CO and CO_2_ was studied, as shown in Figure 7a–d. The CO production relates to the process of incomplete combustion which results in smoldering. As a result, the CO production for the flame retarded RPUF occurs a few seconds earlier, but is insignificantly higher than the pristine RPUF. For instance, the increases in peak of CO production rate (PCO PR) for RPUF-5 and RPUF-6 are 9.4% and 1.9%, respectively, while that of RPUF-7 is 94.3% which is due to the absence of EG, FA, and MP in the constituent composite system. Similarly, the total CO yield (See Figure 7b) increases slightly for all the FRCs except RPUF-6, which is due to the reason that higher loading-level MP induces the formation of intumescent char as observed in Figure 4a,b. The intumescent char creates a barrier effect to prevent the escape of CO from the system.

In contrast to PCO PR, peak CO_2_ production is a result of complete combustion, and therefore, the CO_2_ production for the pristine RPUF is much higher than the flame retarded RPUFs. The values of CO_2_ PR for RPUF-5 and RPUF-6 are reduced by 51.2% and 57.1%, respectively. However, the decline in total CO_2_ yield for the flame retarded RPUF composites is not significant except RPUF-6 for which the total CO_2_ yield is decreased by 23.4%. This phenomenon indicates the potential ability of RPUF-6 to reduce the incident of CO_2_ production of coal associated with coal combustion.

**Figure 8:**
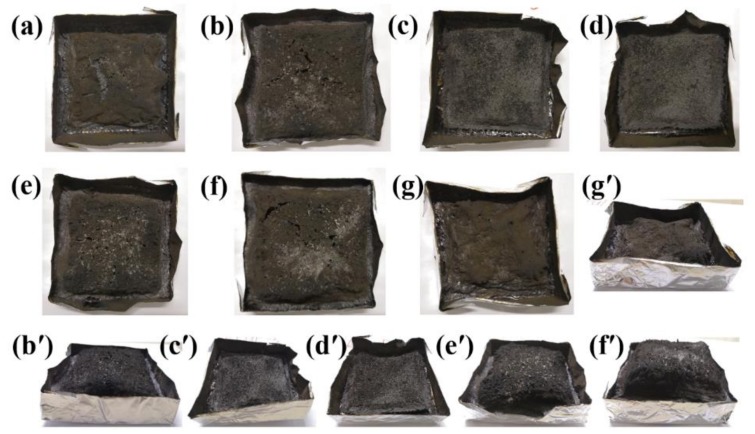
Digital photos of char residues of (**a**) RPUF-1, (**b**,**b′**) RPUF-2, (**c**,**c′**) RPUF-3, (**d**,**d′**) RPUF-4, (**e**,**e′**) RPUF-5, (**f**,**f′**) RPUF-6, and (**g**,**g′**) RPUF-7 after cone calorimeter test.

The digital photos of pristine RPUF and flame retarded RPUF were obtained after the forced flame combustion test, as presented in Figure 8. Pristine RPUF shows scantly weak and porous char residue, while the RPUF-2 presents significantly higher char residue with similar porous structure to RPUF-1. The char residues of RPUF-3, RPUF-4, and RPUF-5 exhibit intumescent and expanded characteristics. The surfaces of RPUF-3 and RPUF-4 are much more compact and smooth in comparison with RPUF-5. Char layers for RPUF-2, RPUF-5, and RPUF-6 are found to have some degree of swelling (See Figure 8b′,e′) according to the height of the char residues, compared to those of RPUF-3 and RPUF-4, indicating the ability of MP to induce intumescence and promote char expansion. Moreover, it is interesting that the degree of expansion is proportional to the loading of MP in RPUF. The degree of char expansion/swelling relates to the quality of the multi-cellular charred structure layer during combustion. With the presence of EG and FA in the char structure, the multi-cellular char layer is strengthened to avoid structural collapses and thus enhance fire safety of the RPUF composites, especially for RPUF-6.

**Figure 9:**
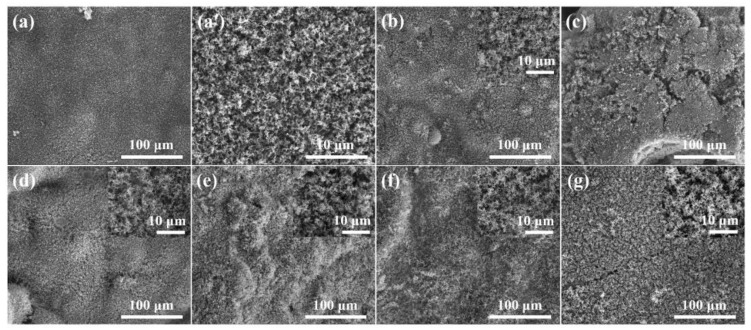
SEM images of external char residues of (**a**,**a′**) RPUF-1, (**b**) RPUF-2, (**c**) RPUF-3, (**d**) RPUF-4, (**e**) RPUF-5, (**f**) RPUF-6, and (**g**) RPUF-7 after cone calorimeter test.

To gain an insight into the structure of residual char structure and how it affects the flame retardancy and gases/smoke performance of the RPUF composites, the external char structure was dissected by SEM, as portrayed in Figure 9. No significant difference can be observed between the pristine RPUF and its FRCs except the seemingly irregular surface morphology of the FRCs. It can be seen that all the RPUF samples show polyporous char surface structure. In addition, the char residues of pristine RPUF and RPUF-2, RPUF-4, RPUF-5, and RPUF-6 maintain integrality with no obvious cracks or holes, whereas RPUF-3 and RPUF-7 exhibit more obvious micro-size char cracks which can serve as channels for the escape of volatile gases and smoke.

**Figure 10:**
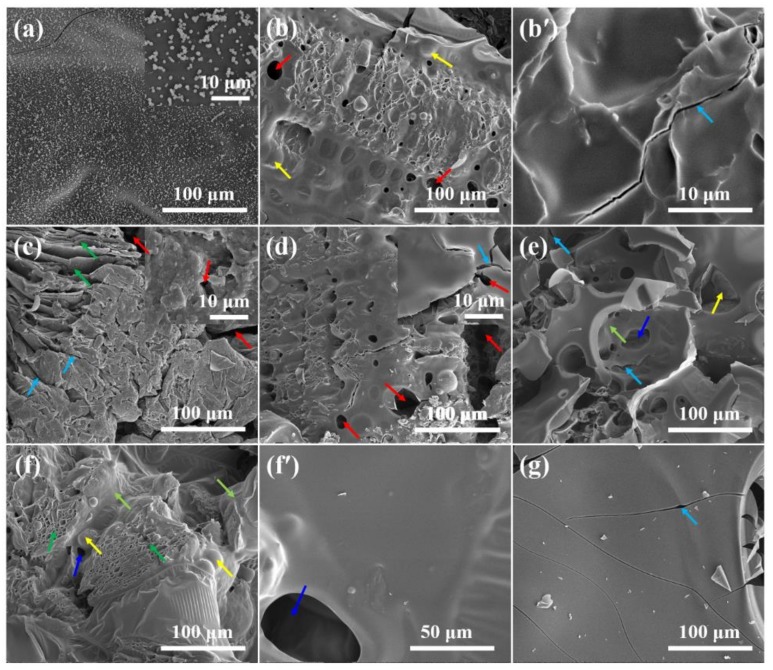
SEM images of internal char residues of (**a**) RPUF-1, (**b**,**b′**) RPUF-2, (**c**) RPUF-3, (**d**) RPUF-4, (**e**) RPUF-5, (**f**,**f′**) RPUF-6, and (**g**) RPUF-7 after cone calorimeter test.

To better understand the effect of morphology of char residues on the flame retardancy of RPUF, the internal char residues of RPUF composites after cone calorimeter tests were also investigated by SEM, as presented in Figure 10. The pristine RPUF show undulating char surface structure (See inserted image in Figure 10a). However, the char is weak due to infinitesimal cracked lines appearing at some spots. RPUF-2 has obvious cracks and microchannel (or holes) companied by quite robust char structure. As the content of EG increases, the expanded ′worm-like′ EG layered structure becomes more apparent with less intumescent materials to cover the expanded layered EG tracks, leading to the formation of more obvious micro-channels and holes. The microchannel tracks and pores serve as tunnels for the exchange of volatiles gases and oxygen in the flaming area, resulting in lower flame-retardant efficiency. Therefore, it is not surprising that both RPUF-3 and RPUF-4 have higher CO, CO_2_, and TSR, although the HRR and THR are marginally lower than those of pristine RPUF. However, it can be clearly observed from Figure 10e that with the increase of MP loading and decline of EG content, the char becomes more compact with shallow nano-size holes. With further increase in MP and subsequent reduction in EG, a more compact and continuous char with sealed EG layers and fewer cavities can be found (See Figure 10f,f′). The random cavities developed are due to the excessive gas-phase phenomenon of RPUF. Furthermore, the continuously smooth and compact char formed by RPUF-6 is largely due to the condensed phase mechanism of MP reinforced by the expansion EG and the foaming predisposition of FA. In contrast, RPUF-7 shows fragile thin char layer with many cracks due to the absence of EG, FA, and MP. The thin nature of the char layer together with the associated cracks is detrimental to the flame-retardant performance and smoke/toxic gases release of RPUF composites. This supposition is confirmed by the analysis of vertical burning and cone calorimeter tests.

**Figure 11:**
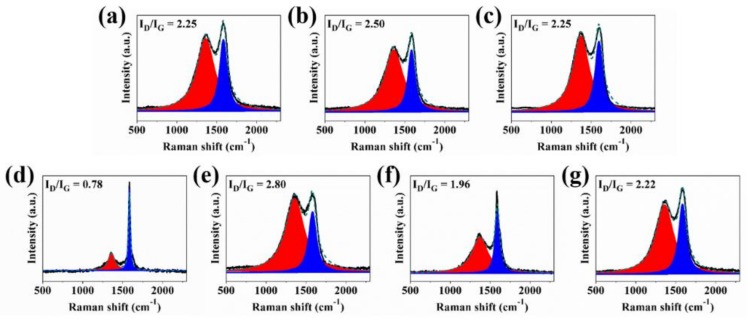
Raman spectra of internal char residues for (**a**) RPUF-1, (**b**) RPUF-2, (**c**) RPUF-3, (**d**) RPUF-4, (**e**) RPUF-5, (**f**) RPUF-6, and (**g**) RPUF-7 after cone calorimeter test.

To analyze the microstructure of char residues, the Raman spectra of the internal and the external structure of char residues for RPUF samples are performed, as plotted in Figure S1 and Figure 11. The characteristic area ratio of D (located at ca. 1360 cm^−1^) and G (situated at around 1580 cm^−1^) bands relates to the degree of graphitization where low I_D_/I_G_ values suggest the abundance of graphitized char [17,44,45,46,47]. Graphitized char is generally thermally stable and reduces the possibility of structural collapses especially for intumescent systems. It is observed from Figure S1 that the I_D_/I_G_ values of the external residual char of RPUF decline gradually with the introduction of CEPPG, EG, FA, and MP at various loadings. For RPUF-6, the I_D_/I_G_ value of the external residual char increases to 2.70, higher than that of RPUF-1 (2.47), whereas the RPUF-7 shows the I_D_/I_G_ value of 2.40, almost close to that of pristine RPUF. The minimal reduction is consistent with the loading of CEPPG in the composites of RPUF-7. However, the fact that RPUF-6 shows the best fire safety among all the samples suggests less contribution of external char structure to enhanced flame retardancy of RPUF.

To well investigate the influence of char residue structure on flame-retardant property of the RPUF, the morphology of internal residual char for all the FRCs was portrayed in Figure 11. Analogously, the I_D_/I_G_ values of internal residual char for RPUF-2, RPUF-3, and RPUF-4 reduce gradually with varying loadings of EG and MP. However, the tendency of I_D_/I_G_ values of the internal residual char for RPUF-5 and RPUF-6 is directly opposite to that observed in the external residual char. It is found that RPUF-6 shows the lowest I_D_/I_G_ values among all the RPUF samples. The phenomenon indicates that the internal residual char contains more graphitized carbons and has the strong compact swollen structure which has been demonstrated in Figure 8f′.

**Figure 12:**
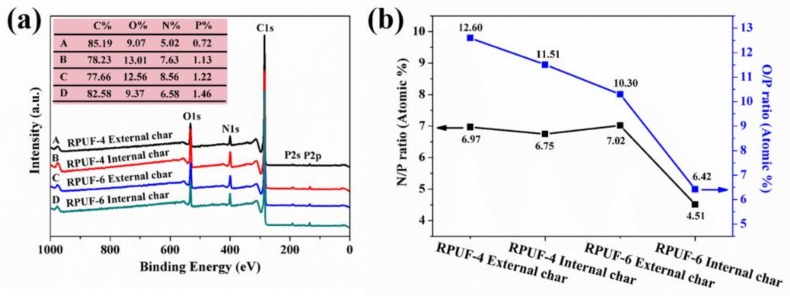
(**a**) XPS spectra and (**b**) N/P ratio and O/P ratio curves of the char residues for RPUF-4 and RPUF-6.

Figure 12a shows the wide scan XPS spectra of the external and the internal residual char of RPUF-4 and RPUF-6. There is much difference between the atomic concentration of these elements in the external and the internal char (See inserted image in Figure 12a). The concentration of carbon (C) in the external char for RPUF-4 is higher than that in the internal char, whereas the concentration of oxygen (O), N, and P elements in the internal char is higher than those in the external char. For RPUF-6, the external char has lower concentration of C and P, and higher the concentration of O and N, in comparison with the internal residual char. Similarly, the atomic ratios of N/P and O/P in RPUF-4 and RPUF-6 are determined from the XPS quantitative analysis, as plotted in Figure 12b. It is found that both N/P ratio and O/P ratio of the internal char residue are markedly lower than those of the external char residue for RPUF-4 and RPUF-6. Moreover, the lowest N/P ratio and O/P ratio are obtained from the internal char residue of RPUF-6 among the char residues of FRCs. The results imply that the internal char residue with high P content contributes greatly to improved flame retardancy of RPUF, which is well accordant with the results of Raman spectra.

**Figure 13:**
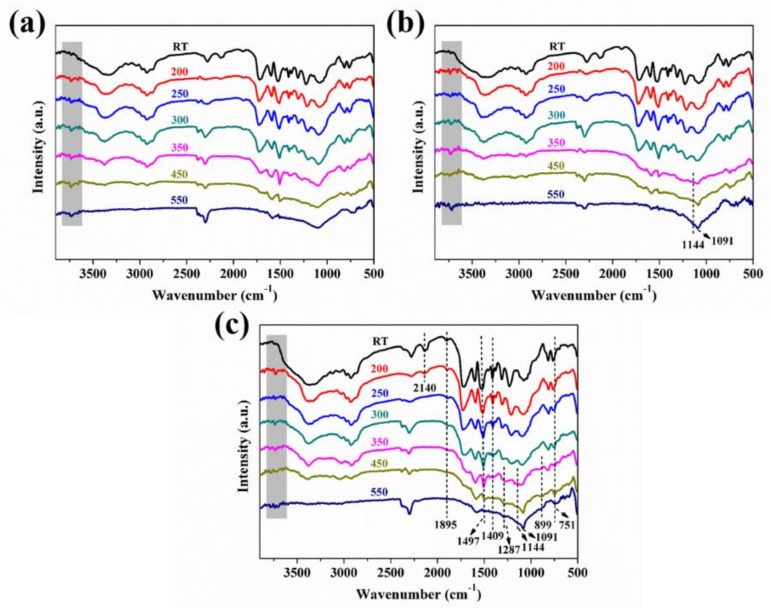
RTIR spectra of (**a**) RPUF-1, (**b**) RPUF-4 and (**c**) RPUF-6.

RTIR spectroscopy is a powerful technique that can monitor the thermal decomposition of materials to identify bond breakages and the formation of new species with increasing thermal energy [48,49,50]. The thermal degradation behavior at different temperature of pristine RPUF, RPUF-4, and RPUF-6 was studied by RTIR, as shown in Figure 13. It can be seen from Figure 13a that below 350 °C, the signal characteristic of the stretching vibrations of N–H groups (in urethane linkages) appears in the range of 3630–3690 cm^−1^ with their bending vibrations observed around 1510–1522 cm^−1^ for pure RPUF [51]. Absorptions corresponding to the stretching vibrations of C=O bonds occur at 1705–1715 cm^−1^ while the stretching vibrations associated with C–N and C–O bonds in urethane linkages appear at 1200–1215 cm^−1^, which is in agreement with the prior work [41]. The symmetric and asymmetric stretching vibrations of C–H bonds occur around 2860–2870 and 2960–2975 cm^−1^. The peaks located at 2260–2280 cm^−1^ are assigned to unreacted N–CO groups, probably due to the excess isocyanate usually needed for the successful manufacturing of foams [41]. The presence of isocyanurate rings and the products of isocyanate trimerization can be observed around 1497–1415 cm^−1^ while the bands around 899–630 cm^−1^ are associated with δ bonds between C and O atoms existing in the ether bonds of polyols. The bonds above remain thermally stable until 350 °C when the intensity of stretching vibrations assigned to C=O and C–N bonds in urethane linkages around 1705–1715 and 1200–1215 cm^−1^ decreases. The stretching vibrations associated with symmetric and asymmetric stretching vibrations of C–H bonds in C–H_2_ bonds in aliphatic chains also disappear. At 550 °C, isocyanurate rings and the products of isocyanate trimerization together with the N–H vibrations in RPUF-1 disappear completely, indicating the total degradation of those linkage bonds.

RPUF-4 shows the similar decomposition behavior to RPUF-1 except the new peaks at the maximum decomposition temperature (550 °C) associated with P–O–C and P–N–C vibrations around 1144 and 1091 cm^−1^, which confirms the presence of phosphorus species in the char residue. It can be seen from Figure 13c that in addition to these peaks assigned to P–O–C and P–N–C groups, the peaks corresponding to the N–CO, C–O groups and the phosphorus species can still be found at 550 °C for RPUF-6, indicating the incomplete combustion of RPUF-6 due to higher MP loading.

### 3.3. Flame-Retardant Mode of Action

**Figure 14:**
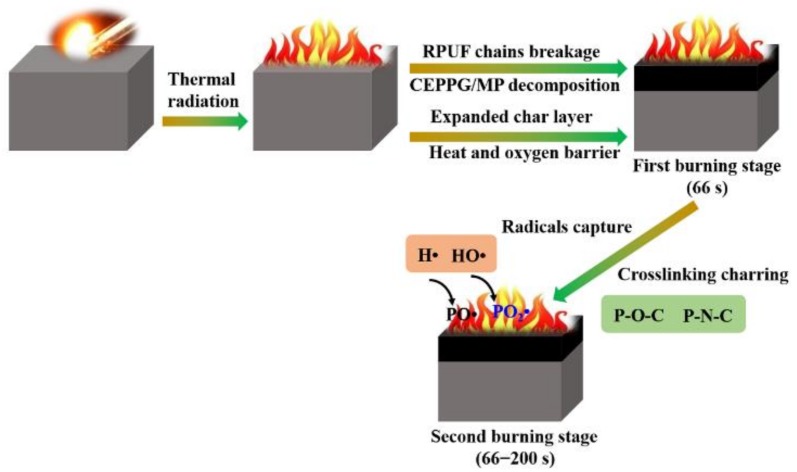
Schematic illustration for the proposed flame-retardant mechanism of RPUF-6.

Based on the results obtained from the UL-94 test, forced flame combustion and the residual char analysis, the flame-retardant mechanism of RPUF composites is proposed in Figure 14. Upon introducing flame/ignition source, dissociation of the thermally instable chains in RPUF into biuret and allophanate occurs [52]. During this process, all the units that connect polyether chains regenerate into their respective precursor compounds made up of isocyanate, hydroxyl compounds, and various forms of amines. At the same time, the decomposition reaction of CEPPG and MP occurs at relatively low temperature and thus releases NO_x_, NH_3_, H_2_O, and PO• radicals which is beneficial to gas-phase flame-retardant action. The evolved incombustible gases (NO_x_, NH_3_ and CO_2_) can dilute the oxygen, leading to reduced degradation rate of RPUF and eventual self-extinguishment after a few minutes. The gas-phase phenomena together with the EG acting as a char expander and MP as swelling agent create effective barriers on the surface of the FRCs to protect the inner foam from further combustion.

The enhanced flame retardancy of RPUF composites during the forced flame combustion is probably attributed to radical trapping by PO• and PO_2_• radicals in the gas phase and more especially intumescent barriers in the condensed phase. During heating process, the gas-phase action becomes obvious by the generation of PO• and PO_2_• radicals and incombustible gases, such as NO_x_, NH_3_ and CO_2_; on the other hand, the EG begins to expand and the MP swells up exponentially. The P-containing radicals act as inert diluent gases to inhibit the spontaneous ignition of the RPUF. Once the RPUF is ignited, the condensed phase mechanism by the P– and N-containing moieties is enhanced by the production of P-containing char network (P–O–C and P–N–C) with the expanded EG serving as a sealant. The thermally stable swollen and sealed char network blocks the entry of oxygen into the flame zone, and simultaneously reduces the release of smoke and gas products generated under the expanded char network (the barrier effect). Though the external char is still exposed to oxygen supply from the ambient environment and the thermal flux from the cone device, the internal char protects RPUF composites from oxygen and heat by the swollen compact thermally stable intumescent char. Hence, the underlying RPUF composites substrate is significantly sheltered from further thermal degradation by the intumescent char.

### 3.4. Retarding Function of Coal Combustion

**Figure 15:**
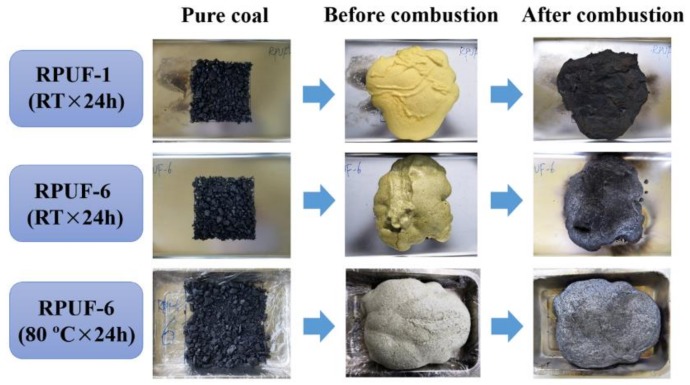
Retarding experiment of coal combustion.

To evaluate the ability of RPUF to prevent the spontaneous combustion of coal, 200 g of coal sample was put in a stainless steel medical square plate with the size of 16 × 16 cm^2^ and thereafter covered by jetting desired amount of pristine RPUF or RPUF-6 composites. Then, the spray gun filled with butane gas was used to cauterize the RPUF composite coal samples for 5 min under different conditions. The obtained results are described in Figure 15. After dried at room temperature for 24 h, the pristine RPUF presents a porously weak char covering on the surface of the coal, and the porous char shows obvious macro channels and cracks which allow oxygen diffusion to support the continuous burning of the coal. Therefore, part of the coal underneath pristine RPUF can be ignited. For RPUF-6, it is easy to observe a more compact and smooth char which reduces the temperature of ignition source, thermal radiation, and the amount of CO generated after dried at room temperature for 24 h. After RPUF-6 is dried at 80 °C for 24 h, a color change and a gargantuan swelling is observed. It is noted that the coal underneath RPUF-6 cannot be ignited after cauterization. The coating forms a dense char layer at the surface of the coal subjected to heat, which prevents oxygen from getting to the surface of the coal. Combustion occurs in the gas phase above the surface of the burning coal. The primary function of the char layer is to act as an insulating barrier to prevent heat feedback to the coal surface. Consequently, coal pyrolysis and consequent formation of volatile fuel fragments are slowed down. The results indicate that the flame retarded RPUF manufactured with a proper loading of CEPPG, EG, MP, and FA is highly effective for reducing the spontaneous fire hazard associated with coal combustion.

## 4. Conclusions

In this study, highly effective flame retarded RPUF composites were designed to inhibit the spontaneous combustion associated with coal mines via using CEPPA and MP as P–N-based FRs and EG as char expander/sealant with FA as foaming agent. The flame retarded RPUF composites containing MP, EG and FA passed the UL-94 V-0 rating whereas the RPUF composite containing CEPPG alone achieved the UL-94 V-1 rating. Significant reductions in PHRR and THR were also attained in addition to substantial decrease in CO and CO_2_ produced for the flame retarded RPUF composites. The flame retarded RPUF reduced the fire hazards associated with the extemporaneous combustion of coal by forming a completely sealed intumescent char layer on the surface of coal to block oxygen access to the coal. Detailed residual char analysis revealed significant contribution of the internal intumescent char. The remarkably enhanced flame retardancy is attributed to char expansion/sealant effect and the condensed phase char inducement predisposition of EG and MP respectively besides the radical trapping effect of the radicals generated by FRs. This work provides an effective and economical means of inhibiting coal spontaneous combustion by manufacturing highly efficient flame-retardant RPUF composites.

## Figures and Tables

**Table 1:** Formulations of RPUF and its composites.

Sample No.	RPUF-1	RPUF-2	RPUF-3	RPUF-4	RPUF-5	RPUF-6	RPUF-7
LY4110 (g)	100.00	77.47	77.47	77.47	77.47	77.47	77.47
CEPPG (g)	0.00	22.53	22.53	22.53	22.53	22.53	22.53
A33 (g)	1.00	1.00	1.00	1.00	1.00	1.00	1.00
LC (g)	0.50	0.50	0.50	0.50	0.50	0.50	0.50
Water (g)	2.00	2.00	2.00	2.00	2.00	2.00	2.00
Si–Oil (g)	2.00	2.00	2.00	2.00	2.00	2.00	2.00
TEOA (g)	3.00	3.00	3.00	3.00	3.00	3.00	3.00
PM-200 (g)	151.66	149.12	149.12	149.12	149.12	149.12	149.12
EG (g)	0.00	13.50	18.00	20.25	9.00	6.75	0.00
MP (g)	0.00	13.50	9.00	6.75	18.00	20.25	0.00
Fly ash (g)	0.00	7.50	7.50	7.50	7.50	7.50	0.00
NCO index	1.10	1.10	1.10	1.10	1.10	1.10	1.10
P wt %	0.00	1.46%	1.25%	1.14%	1.68%	1.78%	0.82%

**Table 2:** The related thermal data of RPUF and its composites under air condition.

Sample No.	T_−5_ (°C)	T_−50_ (°C)	T_max_ (°C)		Residue (wt %)
			Step 1	Step 2	
RPUF-1	249.9	384.5	297.8	585.8	1.99
RPUF-2	232.2	502.9	296.7	559.3	10.34
RPUF-3	246.9	513.2	302.7	560.5	11.68
RPUF-4	252.1	519.0	305.7	566.7	11.96
RPUF-5	241.2	496.8	301.6	565.5	9.97
RPUF-6	246.8	497.4	301.2	576.5	10.51
RPUF-7	253.1	484.8	309.3	584.4	2.40

Notes: T_−5_ means the temperature at 5% weight loss; T_−50_ denotes the temperature at 50% weight loss; T_max_ is the temperature at the maximum weight loss rate.

**Table 3:** The cone calorimeter data of RPUF and its composites at a heat flux of 35 kW m^−2^.

Sample No.	TTI (s)	PHRR (kW m^−2^)	THR (MJ m^−2^)	PSPR (m^2^ s^−1^)	TSR (m^2^ m^−2^)	PCO PR (g s^−1^)	PCO_2_ PR (g s^−1^)	CO TY (kg kg^−1^)	CO_2_ TY (kg kg^−1^)
RPUF-1	4	339	25.4	0.285	5306	0.0053	0.168	0.38	8.76
RPUF-2	5	183	22.3	0.275	4389	0.0069	0.079	0.51	8.46
RPUF-3	4	221	24.2	0.354	6217	0.0076	0.095	1.02	9.14
RPUF-4	5	235	24.6	0.348	5656	0.0075	0.100	1.10	9.80
RPUF-5	4	195	24.1	0.268	4401	0.0058	0.082	0.54	9.29
RPUF-6	5	160	16.3	0.268	3585	0.0054	0.072	0.31	6.66
RPUF-7	6	289	23.3	0.410	5570	0.0103	0.127	0.73	8.68

Notes: TTI—Time to ignition; PHRR—Peak of heat release rate (HRR); THR—Total heat release; PSPR—Peak of smoke production rate (SPR); PCO PR—Peak of CO production rate; PCO_2_ PR—Peak of CO_2_ production rate; CO TY—Total CO yield; CO_2_ TY—Total CO_2_ yield.

## Supplementary Materials

The following are available online at https://www.mdpi.com/2073-4360/11/11/1776/s1. Figure S1: Raman spectra of external char residues of (a) RPUF-1, (b) RPUF-2, (c) RPUF-3, (d) RPUF-4, (e) RPUF-5, (f) RPUF-6, and (g) RPUF-7 after cone calorimeter testing, Table S1: The vertical burning data of RPUF and its composites.
