# Supplementary material for: Highly Effective Flame-Retardant Rigid Polyurethane Foams: Fabrication and Applications in Inhibition of Coal Combustion
Source: Polymers (Basel). 2019 Oct 29;11(11):1776. doi: 10.3390/polym11111776 (PMC6918278; doi:10.3390/polym11111776)
Supplement: Supplementary file 1 [file polymers-11-01776-s001.pdf]

polymers

MDPI

Supplementary Information for

Highly Effective Flame-Retardant Rigid Polyurethane Foams: Fabrication and Applications in Inhibition of Coal Combustion

Liancong Wang [1,5,†], Benjamin Tawiah [3,†], Yongqian Shi [2,*], Suncheng Cai [2], Xiaohui Rao [2], Chuan Liu [2], Ye Yang [2], Fuqiang Yang [2], Bin Yu [4,*], Yuntao Liang [5,*] and Libi Fu [6]

[1] State Key Laboratory of Fire Science, University of Science and Technology of China, 96 Jinzhai Road, Hefei 230026, China; wangliancong-0829@163.com
[2] College of Environment and Resources, Fuzhou University, 2 Xueyuan Road, Fuzhou 350116, China; dandy_cai@163.com (S.C.); Rxh123456789m88@163.com (X.R.); liuchuanll@163.com (C.L.); ys19750873@163.com (Y.Y.); fqouyang82@163.com (F.Y.)
[3] Institute of Textiles and Clothing, Hong Kong Polytechnic University, Hong Kong 999077, China; tawiahb@gmail.com
[4] Centre for Future Materials, University of Southern Queensland, Toowoomba QLD 4350, Australia
[5] State Key Laboratory of Coal Mine Safety Technology, CCTEG Shenyang Research Institute, Fushun 113122, China
[6] College of Civil Engineering, Fuzhou University, 2 Xueyuan Road, Fuzhou 350116, China; fulibi@fzu.edu.cn
† These authors contributed equally to this work (co-first author)
* Corresponding authors: shiyq1986@fzu.edu.cn (Y.S.); ahu07yb@gmail.com (B.Y.); liangyt@fsccri.com (Y.L.)

Received: 29 September 2019; Accepted: 24 October 2019; Published: date

Additional Supporting Data

Table S1. The vertical burning data of RPUF and its composites.

Sample No.	UL-94		
	**t1/t2 (s)**	**Absorbent cotton**	**Rating**
RPUF-1	—	NC	NR
RPUF-2	2.4/1.5	NC	V-0
RPUF-3	1.7/3.7	NC	V-0
RPUF-4	1.7/4.0	NC	V-0
RPUF-5	2.1/0.0	NC	V-0
RPUF-6	1.0/0.0	NC	V-0
RPUF-7	25.5/0.0	NC	V-1

Note: NC indicates no combustion; NR means no rating.

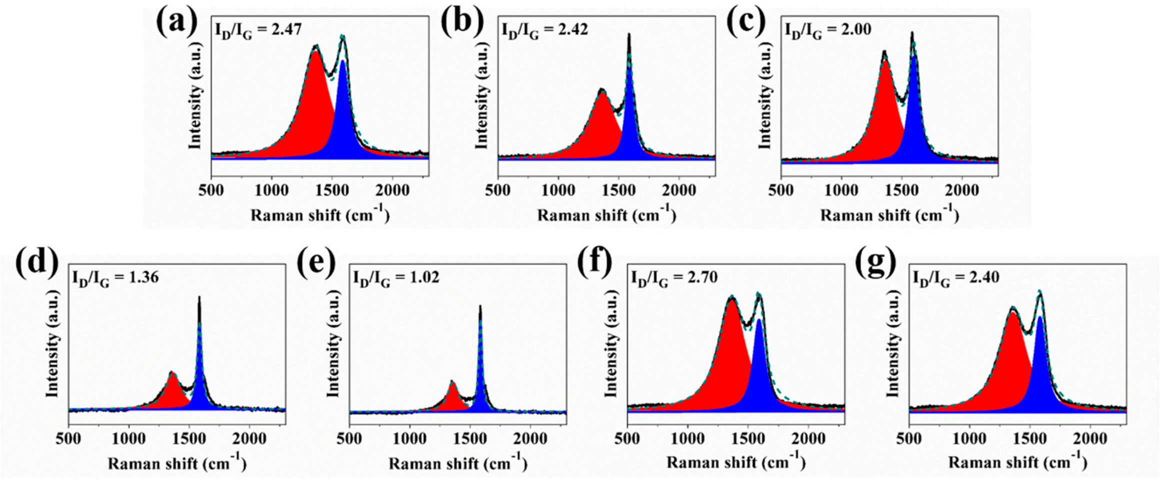

Figure S1. Raman spectra of external char residues of (a) RPUF-1, (b) RPUF-2, (c) RPUF-3, (d) RPUF-4, (e) RPUF-5, (f) RPUF-6, and (g) RPUF-7 after cone calorimeter testing.

© 2019 by the authors. Submitted for possible open access publication under the terms and conditions of the Creative Commons Attribution (CC BY) license (http://creativecommons.org/licenses/by/4.0/).
